# Comparative evaluation of relative fat mass and body mass index in predicting cardiometabolic multimorbidity in older adults: results from the English Longitudinal Study of Ageing

**DOI:** 10.1007/s11357-025-01954-6

**Published:** 2025-10-22

**Authors:** Setor K. Kunutsor, Sae Young Jae, Richard S. Dey, Jari A. Laukkanen

**Affiliations:** 1 Section of Cardiology, Department of Internal Medicine, Rady Faculty of Health Sciences, Max Rady College of Medicine, University of Manitoba, Winnipeg, MB R2H 2A6 Canada; 2 Leicester Real World Evidence Unit, Diabetes Research Centre, University of Leicester, Leicester General Hospital, Gwendolen Road, Leicester, LE5 4WP UK; 3 Department of Sport Science, University of Seoul, Seoul, Republic of Korea; 4 Department of Medicine, University of Ghana Hospital, Legon, Ghana; 5 Institute of Clinical Medicine, Department of Medicine, University of Eastern Finland, Kuopio, Finland; 6 Department of Medicine, Wellbeing Services County of Central Finland, Jyväskylä, Finland

**Keywords:** Relative fat mass, Body mass index, Cardiometabolic multimorbidity, Cohort study

## Abstract

Relative fat mass (RFM) is a more accurate measure of body fat percentage than body mass index (BMI). However, its association with cardiometabolic multimorbidity (CMM) and its predictive value have not been examined. This study evaluated and compared the associations and predictive utility of RFM and BMI for CMM. We analyzed data from 3,348 adults (mean age 64 years; 45.1% male) in the English Longitudinal Study of Ageing who were free of hypertension, coronary heart disease, diabetes, and stroke at wave 4 (2008–2009). RFM was derived from height and waist circumference. CMM was defined at wave 10 (2021–2023) as the presence of two or more of hypertension, cardiovascular disease, diabetes, or stroke. Odds ratios (ORs) with 95% confidence intervals (CIs) and measures of discrimination were estimated. Over 12–15 years of follow-up, 197 participants developed CMM. Restricted cubic spline models showed linear dose–response relationships for both RFM and BMI (*p* for nonlinearity > .05). Higher RFM was strongly associated with CMM (per 1-SD increase: OR 1.66, 95% CI 1.29–2.15; top vs bottom tertile: OR 2.70, 95% CI 1.46–4.99). Associations for BMI were weaker (per 1-SD increase: OR 1.28, 95% CI 1.12–1.47; top vs bottom tertile: OR 1.88, 95% CI 1.24–2.85). Adding RFM to a conventional risk model modestly increased discrimination (ΔC-index = 0.0088, *p* = .29) and significantly improved model fit (-2 log likelihood, *p* < .001). Corresponding values for BMI were ΔC-index = 0.0049 (*p* = .46) and -2 log likelihood (*p* < .001). The C-index gain from RFM was 0.0039 greater than BMI (*p* = .39). In an older UK population, RFM was a stronger indicator and predictor of CMM risk than BMI.

**Supplementary Information:**

The online version contains supplementary material available at https://doi.org/10.1007/s11357-025-01954-6.

## Introduction

Multimorbidity, the coexistence of two or more chronic conditions in an individual, has become an increasingly important challenge in population health as life expectancy improves and populations age [1]. The rising prevalence of multimorbidity contributes substantially to reduced quality of life, increased healthcare utilization, and premature mortality [2, 3]. Among the various constellations of coexisting conditions, cardiometabolic multimorbidity (CMM), commonly defined as the presence of at least two of the following: diabetes mellitus, hypertension, coronary heart disease, and stroke, represents one of the most prevalent and clinically consequential patterns [4]. Cardiometabolic multimorbidity exerts a disproportionate burden on older adults, driving excess morbidity, disability, and healthcare costs [5]. Epidemiological data from diverse populations demonstrate that the prevalence of CMM is increasing globally, paralleling the ongoing epidemics of obesity, sedentary lifestyles, and population ageing [6]. Established risk factors for CMM include unhealthy diet, physical inactivity, smoking, socioeconomic disadvantage, and excess adiposity, many of which are modifiable and therefore attractive targets for prevention [7].

High whole-body fat percentage, reflecting excessive adipose tissue relative to total body weight, is a well-established determinant of adverse cardiometabolic outcomes, including diabetes, hypertension, dyslipidemia, and cardiovascular disease (CVD) [8–10], all of which contribute to CMM. In particular, abdominal obesity, which commonly reflects underlying visceral fat accumulation, is recognized as a major and modifiable contributor to the development of cardiometabolic diseases [8, 11]. Historically, body mass index (BMI) has been widely used as a surrogate for body fatness due to its simplicity. However, BMI has several well-recognized limitations. First, it does not distinguish between fat mass and lean muscle mass [12, 13]. Second, BMI may underestimate risk in individuals who have normal weight but excess visceral fat, and conversely, may paradoxically suggest protective effects in certain groups, such as patients with heart failure – a phenomenon described as the “obesity paradox” [14–16]. Third, BMI often misclassifies adiposity across sex, age, and race/ethnic groups, limiting its utility as a universal measure of obesity-related risk [17]. To address some of these shortcomings, relative fat mass (RFM) was introduced in 2018 by Woolcott and Bergman as a simple, practical, and more accurate index of whole-body fat percentage [18]. Relative fat mass requires only height and waist circumference (WC), thereby incorporating an indicator of central adiposity that is closely linked to cardiometabolic risk. Validation studies have demonstrated that RFM more closely correlates with dual-energy X-ray absorptiometry (DXA)-measured body fat percentage compared with BMI, particularly across diverse ethnic groups [18, 19]. Moreover, higher RFM has consistently been associated with adverse cardiometabolic profiles, including diabetes, hypertension, dyslipidemia, metabolic syndrome, fatty liver disease, increased CVD, and elevated risk of all-cause mortality [20–24]. Importantly, RFM has often been shown to outperform BMI in capturing these associations [21, 22, 24].

Despite growing evidence linking RFM to individual cardiometabolic conditions, there is limited research examining its relationship with CMM. To date, no study has evaluated the association between RFM and incident CMM or assessed the potential predictive utility of RFM for identifying individuals at heightened risk of developing CMM. Addressing this gap is of critical importance, given the rising burden of multimorbidity in ageing populations and the need for simple, reliable tools to aid in risk stratification and prevention. Leveraging data from the English Longitudinal Study of Ageing (ELSA)—a nationally representative, prospective cohort of middle-aged and older adults in England—provides a unique opportunity to investigate these questions. The present study aimed to (i) assess the nature and magnitude of the association between RFM and risk of CMM, (ii) evaluate the predictive utility of RFM for CMM, and (iii) compare these associations with those observed for BMI within the same population sample.

## Methods

### Study population

This study adhered to the STROBE (Strengthening the Reporting of Observational Studies in Epidemiology) guidelines (Supplementary Material 1) [25]. Data were obtained from the ELSA, an ongoing, nationally representative cohort of adults aged 50 years and older living in private households in England [26]. The ELSA sample was initially derived from participants in the 1998, 1999, and 2001 Health Surveys for England [26], forming the foundation for recruitment when the first ELSA wave was launched in 2002–2003 with 11,391 individuals aged ≥ 50 years. Since then, follow-up assessments have been conducted every two years in sequential “waves” [26]. For the present analysis, we defined wave 4 (2008–2009) as baseline and tracked participants through wave 10 (2021–2023). Individuals with pre-existing diagnoses of hypertension, coronary heart disease, diabetes, or stroke at baseline were excluded from the analytic sample [27–29]. We further restricted inclusion to those with complete data on RFM, BMI, covariates, and CMM outcomes. Following application of these criteria, a total of 3,348 participants (men and women) were retained for analysis. All participants provided written informed consent, and ethical approval for the study was granted by the London Multicentre Research Ethics Committee.

### Exposures, covariates and outcome

Information on demographic, lifestyle, and health-related characteristics was obtained through a combination of structured nurse-administered interviews and validated self-completion questionnaires. These tools, which have been widely applied in earlier ELSA investigations, provided data on age, sex, smoking status, alcohol consumption, habitual physical activity, and medical history [26, 27, 30, 31]. Alcohol intake was assessed by asking participants about the frequency of drinking in the past 12 months. Smoking was evaluated in two steps: respondents first indicated whether they had ever smoked (yes/no), and those answering affirmatively were further asked about current smoking status at the time of assessment [31]. Physical activity was captured using a validated instrument that documented participation in light, moderate, and vigorous activities. Following established ELSA protocols, individuals were categorized as sedentary (no moderate or vigorous activity), low (light activity only), moderate (moderate activity at least once weekly), or high (vigorous activity at least once weekly) [26, 30]. Trained nurses conducted standardized health examinations at Mobile Examination Centres, during which anthropometric and functional parameters were measured. These included height, weight, WC, handgrip strength (HGS), and blood pressure, alongside venous blood sampling for biomarker analyses. Body weight was measured to the nearest 0.1 kg using an electronic scale with participants barefoot and lightly clothed, and WC was recorded to the nearest even millimetre at the midpoint between the lower rib margin and the iliac crest [32]. Handgrip strength was determined using a Smedley dynamometer (Stoelting Co., Wood Dale, IL). Participants performed six trials in total, three with each hand, beginning with their dominant side. Hands were alternated with one-minute rest intervals, and the highest value from the six measurements was used in analyses [33]. Body mass index was calculated as weight in kilograms divided by the square of height in meters (kg/m^2^). Relative fat mass was derived using sex-specific equations:: RFM_men_ = 64—[20 x (height/WC)] and RFM_women_ = 76—[20 x (height/WC)], with height and WC expressed in centimeters [18]. Blood pressure was measured on the right arm with an automated Omron HEM 907 monitor (OMRON Healthcare Europe BV, Hoofddorp, the Netherlands). Three readings were obtained, and the mean of the second and third values was used [32]. Chronic conditions, including hypertension, coronary heart disease, diabetes, and stroke, were identified based on participant self-report of physician diagnosis or proxy reporting where applicable [31]. Cardiometabolic multimorbidity was defined at wave 10 as the coexistence of two or more of these conditions: hypertension, CVD, diabetes, or stroke [27–29].

### Statistical analysis

Descriptive statistics were used to summarize baseline participant characteristics. Continuous variables were reported as means with standard deviations (SD) or medians with interquartile ranges (IQR), depending on distributional properties assessed through graphical inspection and tests of normality. Categorical variables were expressed as counts and percentages. Comparisons across groups were evaluated using analysis of variance (ANOVA) for continuous measures and chi-square tests for categorical variables. To investigate the shape of the associations between RFM, BMI, and subsequent CMM, we fitted multivariable logistic regression models incorporating restricted cubic splines (RCS). Knot placement followed Harrell’s guidance for moderately sized datasets, positioned at the 5th, 35th, 65th, and 95th percentiles of each exposure distribution [34]. Departure from linearity was assessed using likelihood ratio tests, contrasting models with only a linear term against those including both linear and spline components [35, 36]. Multivariable logistic regression was then applied to estimate odds ratios (ORs) with 95% confidence intervals (CIs). Three hierarchical models were specified: Model 1 adjusted for age and sex; Model 2 additionally accounted for smoking status, alcohol intake, systolic blood pressure (SBP), total cholesterol, high-density lipoprotein cholesterol (HDL-C), and HGS; and Model 3 further included physical activity. These covariates were chosen given their established relevance to cardiometabolic disease, previous evidence from ELSA analyses [27–30, 33], and their potential role as confounders [37]. Because the RCS results suggested approximately linear relationships, RFM and BMI were modeled as continuous exposures (per 1-SD increment) as well as categorical variables (tertiles). To assess predictive performance, C-indices were estimated for models containing conventional risk factors (age, sex, smoking status, alcohol consumption, SBP, total cholesterol, HDL-C, HGS, and physical activity) with and without the addition of RFM or BMI. The difference in C-indices was tested using DeLong’s method [38]. In recognition of the limitations of discrimination metrics alone, we also compared models using the −2 log likelihood test, which has been recommended as a sensitive approach for evaluating changes in model fit when considering potential biomarkers [39, 40]. All statistical analyses were performed using Stata MP version 18.0 (StataCorp, College Station, TX).

## Results

### Baseline characteristics

Table 1 presents the baseline characteristics of the study's participants overall and by RFM tertiles. The mean (SD) age of the 3,348 study participants at baseline was 63.5 (8.5) years and males constituted 45.1% of the cohort. The mean (SD) of RFM and BMI was 34.9 (6.9) and 27.6 (4.9) kg/m^2^, respectively. Participants with higher RFM (tertile 3) were older and less likely to be male, had higher levels of anthropometric indices (BMI, WC, and weight), less likely to have moderate to high levels of physical activity or consume alcohol frequently, had higher levels of SBP and lipids, and lower HGS.

**Table 1:** Baseline characteristics overall and by categories of RFM

		Relative fat mass			
Characteristic	Overall (N=3348)	Tertile 1 (N=1116)	Tertile 2 (N=1116)	Tertile 3 (N=1116)	*p*-value
	Mean (SD), Median (IQR), or n (%)	Mean (SD), Median (IQR), or n (%)	Mean (SD), Median (IQR), or n (%)	Mean (SD), Median (IQR), or n (%)	
Relative fat mass	34.9 (6.9)	27.3 (2.8)	34.5 (2.3)	42.9 (3.1)	
Body mass index, kg/m^2^	27.6 (4.9)	25.2 (2.9)	27.1 (4.7)	30.4 (5.3)	<.001
Waist circumference, cm	95.0 (13.3)	93.1 (10.3)	93.8 (16.6)	98.1 (11.5)	<.001
Height, cm	166.7 (9.5)	173.9 (7.5)	166.2 (8.3)	159.9 (6.9)	<.001
Weight, kg	76.7 (15.6)	76.5 (12.2)	75.8 (18.6)	77.8 (15.4)	.01
Age, yrs	63.5 (8.5)	63.0 (8.2)	63.4 (8.7)	64.0 (8.7)	.016
Sex					<.001
Male	1510 (45.1%)	1011 (90.6%)	482 (43.2%)	17 (1.5%)	
Female	1838 (54.9%)	105 (9.4%)	634 (56.8%)	1099 (98.5%)	
Ethnicity					
White	3288 (98.3%)	1098 (98.5%)	1100 (98.6%)	1090 (97.8%)	.28
Non-White	58 (1.7%)	17 (1.5%)	16 (1.4%)	25 (2.2%)	
Current smoker					.44
No	2873 (85.8%)	947 (84.9%)	958 (85.8%)	968 (86.7%)	
Yes	475 (14.2%)	169 (15.1%)	158 (14.2%)	148 (13.3%)	
Alcohol categories					<.001
None	1121 (33.5%)	308 (27.6%)	331 (29.7%)	482 (43.2%)	
1–2 times/wk	843 (25.2%)	263 (23.6%)	319 (28.6%)	261 (23.4%)	
3–4 times/wk	636 (19.0%)	232 (20.8%)	230 (20.6%)	174 (15.6%)	
5 or more times/wk	748 (22.3%)	313 (28.0%)	236 (21.1%)	199 (17.8%)	
Handgrip strength, kg	31.0 (11.4)	38.9 (10.0)	30.5 (11.0)	23.6 (6.9)	<.001
Systolic blood pressure, mmHg	130 (17)	130 (16)	130 (17)	132 (17)	<.001
Total cholesterol, mmol/L	5.84 (1.14)	5.61 (1.05)	5.83 (1.16)	6.08 (1.15)	<.001
HDL cholesterol, mmol/L	1.59 (0.42)	1.53 (0.41)	1.61 (0.46)	1.64 (0.37)	<.001
Triglyceride, mmol/L	1.40 (1.00, 2.00)	1.30 (1.00, 1.90)	1.40 (1.00, 2.00)	1.55 (1.10, 2.20)	<.001
PA level					<.001
Sedentary	91 (2.7%)	23 (2.1%)	36 (3.2%)	32 (2.9%)	
Low	593 (17.7%)	108 (9.7%)	187 (16.8%)	298 (26.7%)	
Moderate	1810 (54.1%)	612 (54.8%)	615 (55.1%)	583 (52.2%)	
High	854 (25.5%)	373 (33.4%)	278 (24.9%)	203 (18.2%)	

*HDL* high-density lipoprotein, *IQR* interquartile range, *PA* physical activity, *RFM* relative fat mass, *SD* standard deviation

### Associations of RFM and BMI with CMM

**Fig. 1:**
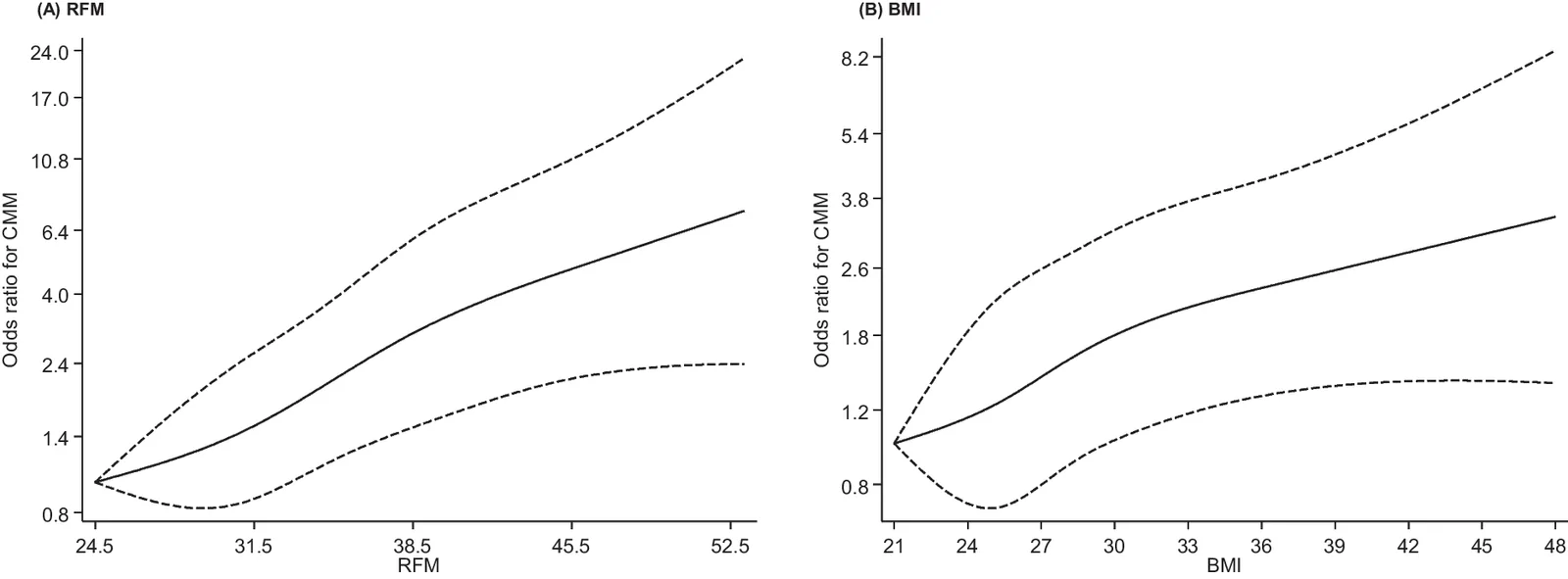
Spline curve of the associations of RFM and BMI with the risk of cardiometabolic multimorbidity. (**A**) RFM (**B**) BMI. BMI, body mass index; RFM, relative fat mass. Dashed lines represent the 95% confidence intervals for the spline model (solid line). The model was adjusted for age, sex, smoking status, alcohol consumption, systolic blood pressure, total cholesterol, high-density lipoprotein cholesterol, handgrip strength, and physical activity

**Fig. 2:**
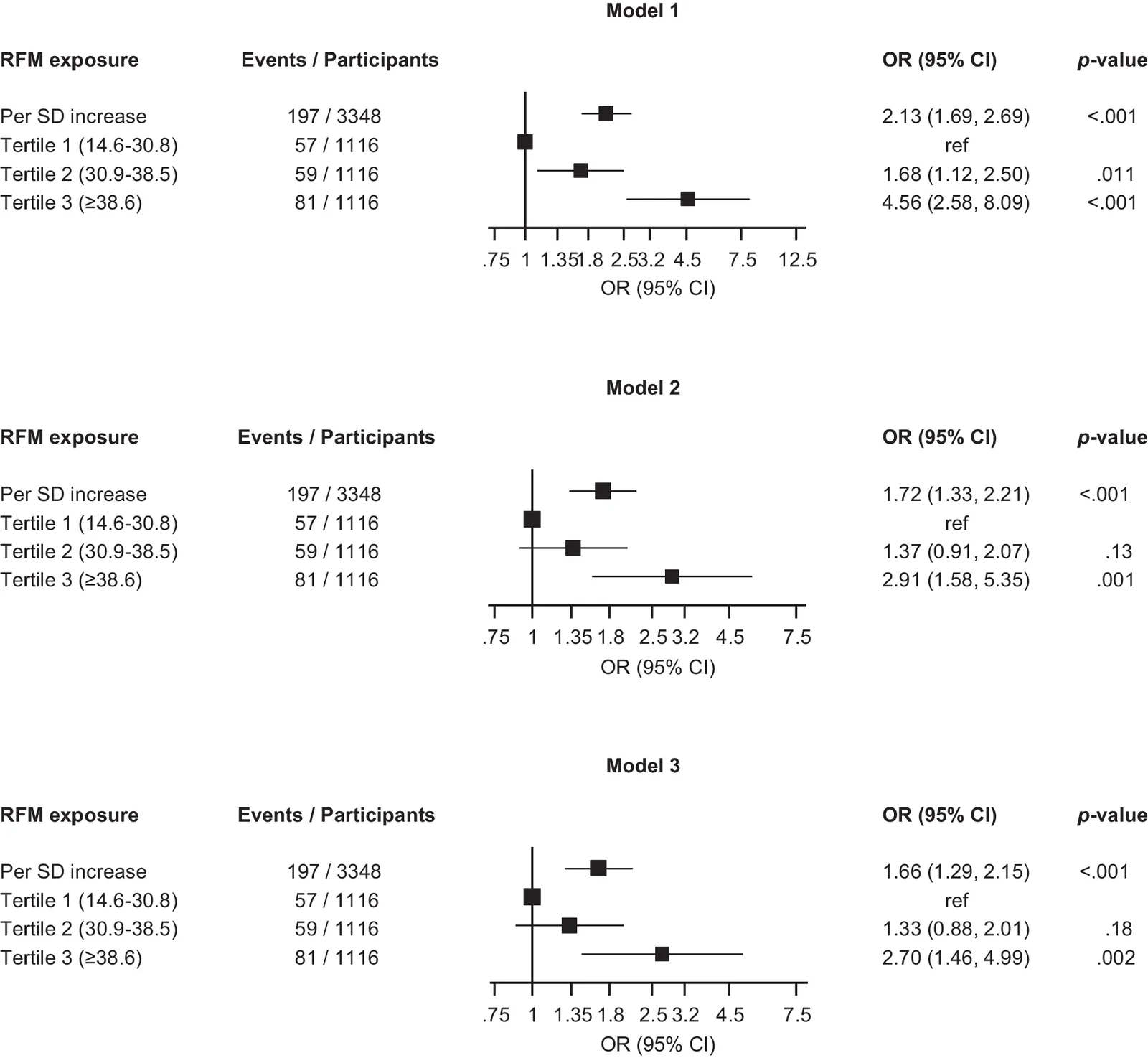
Associations of RFM with cardiometabolic multimorbidity. CI, confidence interval, OR, odds ratio; RFM, relative fat mass; SD, standard deviation. Model 1: Adjusted for age and sex. Model 2: Model 1 plus smoking status, alcohol consumption, systolic blood pressure, total cholesterol, high-density lipoprotein cholesterol, and handgrip strength. Model 3: Model 2 plus physical activity

Over 12–15 years of follow-up, 197 participants developed CMM. Multivariable RCS analyses indicated a linear association between RFM and CMM risk (*p* for nonlinearity = 0.78), with risk rising steadily across the RFM range of 24.5 to 52.5 (Fig. 1A). In analysis adjusted for age, sex, smoking status, alcohol consumption, SBP, total cholesterol, HDL-C, and HGS, the OR for CMM per 1-SD increment in RFM was 1.72 (95% CI: 1.33–2.21) (Fig. 2-Model 2), which was minimally attenuated following further adjustment for physical activity 1.66 (95% CI: 1.29–2.15) (Fig. 2**-**Model 3). Comparing the top vs bottom tertiles of RFM, the corresponding adjusted ORs were 2.91 (95% CI: 1.58–5.35) and 2.70 (95% CI: 1.46–4.99), respectively (Fig. 2-Models 2 and 3).

**Fig. 3:**
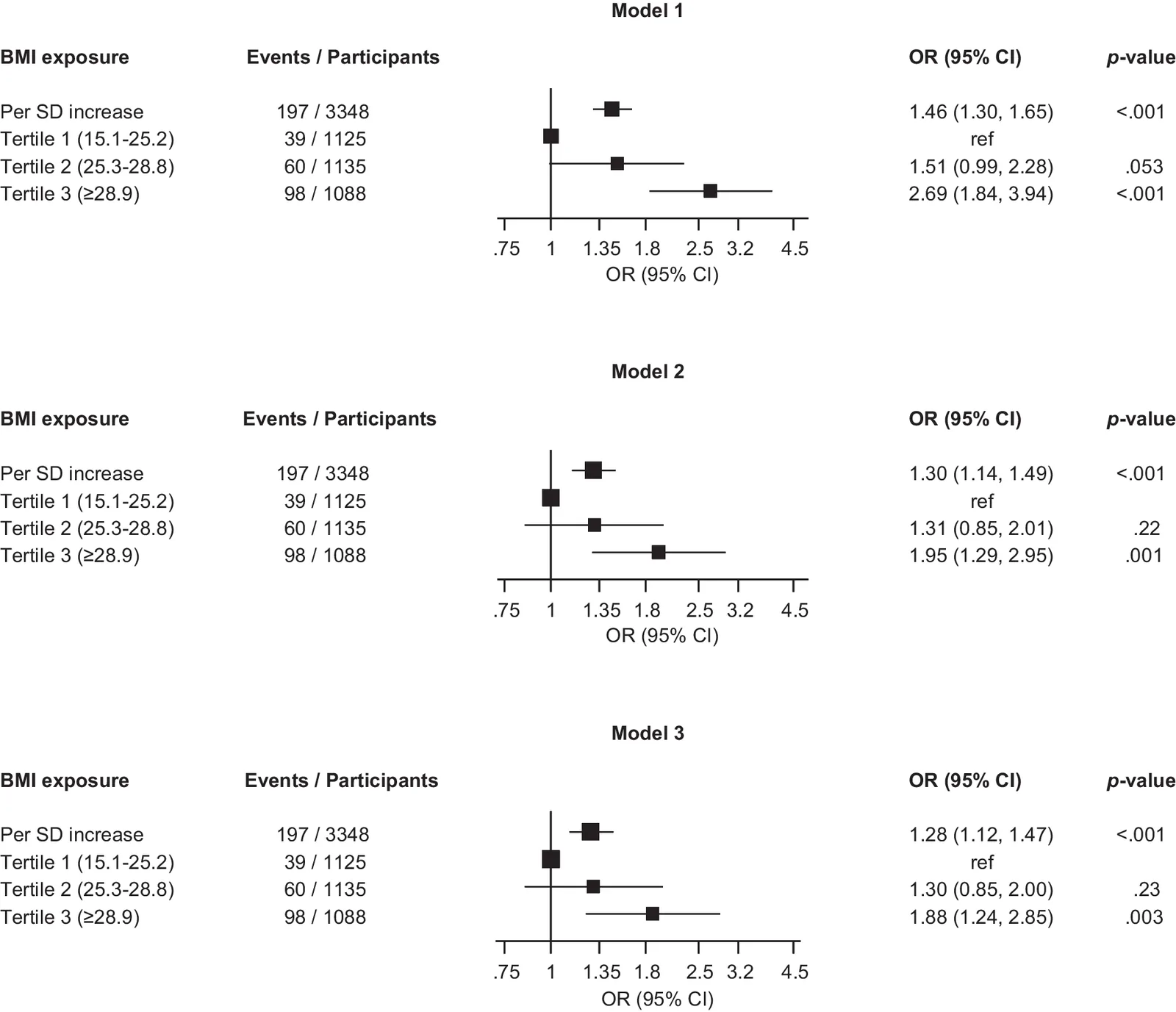
Associations of BMI with cardiometabolic multimorbidity. BMI, body mass index; CI, confidence interval, OR, odds ratio; SD, standard deviation. Model 1: Adjusted for age and sex. Model 2: Model 1 plus smoking status, alcohol consumption, systolic blood pressure, total cholesterol, high-density lipoprotein cholesterol, and handgrip strength. Model 3: Model 2 plus physical activity

For BMI, a similar linear dose–response pattern was observed (*p* for nonlinearity = 0.68), with CMM risk increasing across the BMI range of 21–48 kg/m^2^, although the slope was less steep than for RFM (Fig. 1B). In analysis adjusted for model 2 covariates, the OR for CMM per 1-SD increase in BMI was 1.30 (95% CI: 1.14–1.49) (Fig. 3**-**Model 2), which was minimally attenuated following further adjustment for physical activity 1.28 (95% CI: 1.12–1.47) (Fig. 3-Model 3). Comparing extreme tertiles, the corresponding adjusted ORs were 1.95 (95% CI: 1.29–2.95) and 1.88 (95% CI: 1.24–2.85), respectively (Fig. 3**-**Models 2 and 3).

When comparing effect sizes, the OR for the association between RFM and CMM (OR = 2.70) was substantially stronger than that for BMI (OR = 1.88). This corresponds to a 48% attenuation, calculated as [2.70–1.88)/2.70 × 100], when BMI was used instead of RFM.

### Risk prediction

The results of the risk prediction analyses are summarized in Table 2. A baseline model including established risk factors produced a C-index of 0.6892 (95% CI: 0.6500, 0.7285). Adding RFM increased the C-index to 0.6980 (95% CI: 0.6590, 0.7371), corresponding to a non-significant gain of 0.0088 (*p* = 0.29). Nonetheless, model fit improved significantly with the inclusion of RFM, as indicated by the −2 log likelihood test (*p* < 0.001). For BMI, the corresponding changes were smaller (ΔC-index = 0.0049, *p* = 0.46), though the −2 log likelihood test also showed significant improvement (*p* < 0.001). Overall, the incremental improvement in discrimination achieved with RFM was 0.0039 higher than that observed for BMI, but this difference was not statistically significant (*p* = 0.39).

**Table 2:** Measures of risk discrimination upon addition of RFM and BMI to a CMM risk model containing established risk factors

Measure of discrimination	RFM	BMI
C-index (95% CI): established risk factors	0.6892 (0.6500, 0.7285)	0.6892 (0.6500, 0.7285)
C-index (95% CI): established risk factors plus exposure	0.6980 (0.6590, 0.7371)	0.6941 (0.6551, 0.7331)
C-index change (*p*-value)	0.0088 (.29)	0.0049 (.46)
*p*-value for difference in −2 log likelihood	<.001	<.001

*BMI* body mass index *CI* confidence interval, *CMM* cardiometabolic multimorbidity, *RFM* relative fat mass, established risk factors include age, sex, smoking status, alcohol consumption, systolic blood pressure, total cholesterol, high-density lipoprotein cholesterol; handgrip strength, and physical activity level

## Discussion

In this large, nationally representative cohort of older adults, we found that higher RFM was associated with an adverse baseline cardiometabolic profile, including older age, higher adiposity indices, less favorable lifestyle patterns, elevated blood pressure and lipid levels, and reduced muscle strength. Over a median follow-up of 12–15 years, both RFM and BMI demonstrated linear, dose–response associations with the risk of CMM. However, the magnitude of association was stronger for RFM than for BMI, with participants in the highest tertile of RFM showing nearly three-fold higher odds of developing CMM compared with those in the lowest tertile. In predictive analyses, adding RFM to established risk factors modestly improved model fit and discrimination, with a greater incremental gain compared with BMI, although differences in C-index were not statistically significant. Taken together, these findings suggest that RFM is a stronger risk indicator and potentially more informative predictor of CMM than BMI in older adults.

Previous research has consistently shown that higher RFM is associated with adverse cardiometabolic outcomes, including diabetes, hypertension, dyslipidemia, fatty liver disease, CVD, and increased mortality risk [20–24]. However, to our knowledge, no study has specifically examined the association of RFM with CMM or evaluated the predictive utility of RFM for CMM. The present analysis therefore addresses an important gap in the literature. By demonstrating that RFM is more strongly associated with the risk of developing CMM than BMI and provides incremental predictive value, this study offers novel insights into the potential of RFM as a simple and informative anthropometric tool for identifying individuals at high risk of CMM in older populations.

 The positive association between RFM and CMM likely reflects the detrimental impact of excess visceral and ectopic fat, which RFM was designed to capture through the inclusion of WC. Visceral adiposity plays a central role in the development of dyslipidemia, hypertension, T2D, and CVD through interconnected mechanisms [41, 42]. As adiposity increases, ectopic fat depots initiate a cascade of metabolic disturbances driven by chronic low-grade inflammation, oxidative stress, and insulin resistance, largely mediated by pro-inflammatory cytokines and macrophage infiltration [43]. Fat accumulation in organs such as the liver, pancreas, and skeletal muscle disrupts glucose and lipid regulation, further compounding metabolic dysfunction [44]. Moreover, visceral fat is associated with lower adiponectin levels and leptin resistance, both of which impair insulin sensitivity and metabolic regulation [44, 45]. These processes accelerate lipolysis and increase circulating free fatty acids and inflammatory mediators [46], which in turn promote vascular inflammation, endothelial dysfunction, and coagulation abnormalities—hallmarks of the clustered conditions that define CMM. Relative fat mass provides a stronger risk signal than BMI because it incorporates WC, thereby accounting for central adiposity, which is more directly implicated in these pathogenic pathways. In contrast, BMI does not differentiate between fat and lean mass and fails to capture the distribution of adiposity [12, 13], leading to misclassification and weaker associations with cardiometabolic risk.

The present findings have several important clinical and public health implications. Relative fat mass is as simple to measure as BMI, requiring only height and WC, yet unlike BMI it provides an approximate estimate of body fat percentage. RFM has been shown to correlate more strongly with DXA-measured body fat, offering a practical and cost-effective alternative for clinical and population health settings.

Because RFM captures central adiposity, it may identify individuals with excess body fat who are misclassified as “normal weight” by BMI, thereby improving risk stratification. This is particularly relevant for early identification of individuals at high risk of CMM, enabling more timely lifestyle and therapeutic interventions. Integrating RFM into existing health screening frameworks could enhance the prediction and prevention of cardiometabolic risk in community and clinical settings. Although our results underscore the value of RFM in an older English population, further research is warranted to determine whether its predictive utility holds across different age groups, younger populations, and ethnically diverse cohorts. Moreover, future studies should evaluate whether RFM-based risk screening can effectively inform targeted preventive strategies to reduce the growing burden of CMM. Establishing its consistency and utility across diverse settings would support the broader adoption of RFM as a simple yet informative tool for cardiovascular and metabolic risk assessment.

This study offers several important strengths. To our knowledge, it is the first to prospectively investigate the association between RFM and CMM, as well as to evaluate the added value of RFM in predicting CMM risk compared with BMI. By utilizing data from the ELSA, we were able to generate findings that are both methodologically robust and broadly relevant to real-world populations. The statistical analyses were rigorous, incorporating RCS models to explore dose–response relationships, comprehensive adjustment for established and emerging cardiometabolic risk factors, and formal tests of predictive performance using validated discrimination metrics. Nevertheless, some limitations should be acknowledged. As with all observational analyses, causality cannot be firmly established. Exposures and outcomes, including chronic conditions and lifestyle factors, were primarily self-reported, raising the possibility of misclassification, recall error, or social desirability bias. Although prior validation studies indicate moderate agreement between self-reported and clinically verified diagnoses [47, 48], the lack of medical record confirmation may introduce measurement error. Despite adjusting for a wide range of potential confounders, residual confounding due to errors in measured confounders and relevant unmeasured confounders cannot be excluded. In addition, the absence of diagnosis dates precluded the use of time-to-event models, limiting the assessment of temporal relationships. The cohort was restricted to older English adults, which may reduce generalizability to younger populations and other ethnic groups. A further limitation of this study is the relatively low number of participants who developed CMM during follow-up, which may have reduced the precision of some estimates and limited our ability to explore subgroup effects. Although our findings were derived from a relatively large, nationally representative sample of older adults, replication in larger and more ethnically diverse populations is needed to confirm the robustness and generalizability of these results. Additionally, while we included ethnicity (categorized as White and Non-White) in the baseline characteristics, the small number of participants in the latter group precluded stratified analyses by ethnicity. The findings should be interpreted in the context of the limitations.

## Conclusions

In this nationally representative cohort of older UK adults, both RFM and BMI were positively and linearly associated with the risk of CMM and each provided incremental value in risk prediction beyond established risk factors. Importantly, RFM emerged as a stronger risk indicator and more informative predictor of CMM than BMI, highlighting its potential utility as a simple and clinically relevant tool for identifying individuals at heightened cardiometabolic risk.

## Supplementary Information

Below is the link to the electronic supplementary material.

Supplementary file1 (DOCX 21 KB)

## Data Availability

Data from the ELSA are available to the public to download from the UK Data Service at https://ukdataservice.ac.uk/.
